# A multidimensional integration analysis reveals potential bridging targets in the process of colorectal cancer liver metastasis

**DOI:** 10.1371/journal.pone.0178760

**Published:** 2017-06-19

**Authors:** Bo Gao, Tian Yu, Dongbo Xue, Boshi Sun, Qin Shao, Hani Choudhry, Victoria Marcus, Jiannis Ragoussis, Yuguo Zhang, Weihui Zhang, Zu-hua Gao

**Affiliations:** 1 Department of General Surgery, the First Affiliated Hospital of Harbin Medical University, Harbin, China; 2 Department of Pathology, The Research Institute of McGill University Health Center, Montreal, Québec, Canada; 3 Section of Immunity, Infection and Inflammation, Division of Applied Medicine, School of Medicine and Dentistry, Institute of Medical Sciences, University of Aberdeen, Aberdeen, Scotland, United Kingdom; 4 Department of General Surgery, the Second Affiliated Hospital of Harbin Medical University, Harbin, China; 5 Department of Biochemistry, Faculty of Science, Cancer and Mutagenesis Unit, King Fahd Center for Medical Research, Center of Innovation in Personalized Medicine, King Abdulaziz University, Jeddah, Saudi Arabia; 6 McGill University and Genome Quebec Innovation Centre, Montreal, Québec, Canada; 7 Department of Traditional and Western Medical Hepatology, The Third Hospital of Hebei Medical University, Shijiazhuang, China; Universitat des Saarlandes, GERMANY

## Abstract

Approximately 9% of cancer-related deaths are caused by colorectal cancer. Liver metastasis is a major factor for the high colorectal cancer mortality rate. However, the molecular mechanism underlying colorectal cancer liver metastasis remains unclear. Using a global and multidimensional integration approach, we studied sequencing data, protein-protein interactions, and regulation of transcription factor and non-coding RNAs in primary tumor samples and liver metastasis samples to unveil the potential bridging molecules and the regulators that functionally link different stages of colorectal cancer liver metastasis. Primary tumor samples and liver metastasis samples had modules with significant overlap and crosstalk from which we identified several bridging genes (e.g. KNG1 and COX5B), transcription factors (e.g. E2F4 and CDX2), microRNAs (e.g. miR-590-3p and miR-203) and lncRNAs (e.g. lincIRX5 and lincFOXF1) that may play an important role in the process of colorectal cancer liver metastasis. This study enhances our understanding of the genetic alterations and transcriptional regulation that drive the metastatic process, but also provides the methodology to guide the studies on other metastatic cancers.

## Introduction

Colorectal cancer (CRC) is the second most common cancer in men, the third most common cancer in women, and accounts for approximately 9% of all cancer deaths[1]. Liver metastasis is a major factor that is responsible for the high mortality rate seen in patients with CRC. Approximately 30–50% patients either already have liver metastasis at the time of the CRC diagnosis, or will have liver metastasis after radical resection of the primary lesion. Unfortunately, only 10–20% of liver metastatic lesions can be surgically removed[2, 3]. The median survival time of patients with untreated liver metastatic lesions is only 6.9 months, and the 5-year survival rate is close to 0%. Even when the metastatic lesions can be completely resected, the average survival time is only 35 months [3]. Therefore, it is imperative to elucidate the underlying molecular mechanisms of CRC liver metastasis in order to better treat these patients and improve their survival.

Tumor invasion and metastasis are dynamic processes involving multiple steps. These processes primarily include: 1) massive proliferation of tumor cells in primary lesions, 2) the acquirement of metastatic genes by a small population of tumor cells, 3) the detachment of tumor cells from primary lesions, 4) the invasion of the basement membrane, 5) entry into and exit from the circulatory system, and 6) the colonization and formation of secondary tumors at a distant site [4]. Based on the characteristics of tumor metastasis, our group collected samples from CRC patients in different stages of liver metastasis for next generation sequencing. The data were uploaded into the Gene Expression Omnibus (GEO) database (GSE72718).

Over decades of intensive research, several individual functional molecules have been identified in the process of CRC liver metastasis, such as KRAS, BRAF, and EGFR[5, 6]. However, the progression of tumor metastasis likely involves a coordinated effect on multiple biological processes including the differential expression of genes and the abnormal regulation of transcription and translation[7–9]. Therefore, compared with single-line mechanistic studies, a multidimensional integration analysis from a global perspective can help us to comprehensively and accurately understand the mechanisms that underlie CRC liver metastasis.

In this study, we used a global and multidimensional integration strategy to analyze the sequencing data, protein-protein interaction (PPI) and the regulation data of transcription factor (TF) and non-coding RNA from samples obtained from primary non-metastatic colorectal tumor (PNMCT), primary metastatic colorectal tumor (PMCT), and their paired metastatic CRC samples in the liver (LMCT). This approach enabled us to identify the potential bridging molecules that play a role in the process of CRC liver metastasis including modules with significant overlap and crosstalk. Furthermore, some potential molecular targets including TFs, miRNAs, and lncRNAs were identified through the establishment of a regulatory network of modules. Our results from this comprehensive analysis demonstrate a new methodology for studying the molecular mechanisms of CRC liver metastasis.

## Materials and methods

### Sample collection, gene sequencing and identification of differentially expressed genes (DEGs)

This study was approved by GEN (Genetics/Population Research/Investigator Initiated Studies) Search Ethics of McGill University Health Center. (Approval number:14-448GEN(HRR#4226). The patient consent was waived by the ethics committee as our study only used archived tissue and had no to patient care. With the approval of the institutional ethics review board, we collected 10 PNMCT samples, 9 PMCT samples and 9 LMCT samples from 19 patients with CRC who received the diagnosis and treatment in McGill University Health Center. This investigation was conducted according to the Declaration of Helsinki. Affymetrix Human Transcriptome Array 2.0 was used to sequence the gene expression of these samples. The sequencing data was uploaded to the GEO database (No. GSE72718).

Based on these sequencing data, we used the R limma package to identify the DEGs in PMCT (PMCT vs PNMC) and LMCT (LMCT vs PNMCT). The threshold of DEGs is |log_2_Fold Change|>|log_2_1.2| and p value<0.05. Moreover, we established the heatmap of DEGs. Finally, we used the principal component analysis (PCA) method to confirm the correlation between disease and DEGs[10].

### PPI network and module analysis

The STRING database V10 (http://string-db.org/) and Cytoscape software were used to establish the PPI networks of PMCT and LMCT[11]. MCODE, a Cytoscape plugin that finds highly interconnected regions in a network, was used to identify the DEG modules in PPI network[12].

### Exploring modules with significant overlap between PMCT and LMCT

For the PMCT- and LMCT-associated module pair, we computed the significance of their overlapping DEGs using a hypergeometric test as follows: M and n represent the number of genes in PMCT and LMCT modules, respectively. N is the number of genes in the STRING database and m is the number of overlapping DEGs[13]. The threshold of an overlapping module pair was p<0.05.

$$p=1-\sum_{i=0}^{m-1}\frac{\left(\begin{array}{c} n\\ i \end{array})(\begin{array}{c} N-n\\ M-i \end{array}\right)}{\left(\begin{array}{c} N\\ M \end{array}\right)}$$

### Exploring modules with significant crosstalk between PMCT and LMCT

The significance of crosstalk between the sub-network modules of PMCT and LMCT is primarily determined by their interaction times and by comparing results of random computation. One pair of sub-network modules of PMCT and LMCT had m times of participation interaction in actual conditions. The original PPI network was randomized 1000 times by maintaining the degree of distribution of the unchanged nodes. The two sub-network modules with the same size as the original network modules were randomly screened[14]. We computed the interaction times in random sub-network modules in the same pair of PMCT and LMCT modules. The p value for the significance of interaction between a single pair of sub-network modules was calculated as the randomized simulation computation of interaction times larger than the actual participation interaction times divided by 1000 times. Interactive sub-network modules with a p value lower than 0.05 were considered significant interactive sub-networks.

### Establishing regulatory networks of modules with significant overlap and crosstalk between PMCT and LMCT

For the PMCT and LMCT module pair, we determined their regulators (TFs and miRNAs) as: (i) at least two regulations between the regulator and each module of the pair and (ii) significant enrichment of targets for each regulator per module with a p value cutoff of 0.05 [15]. In this study, we used the ChIPBase database to predict TFs, several databases including miRecords, MiRWalk2.0, miRanda, MiRTarget2, PicTar, PITA and TargetScan to predict miRNAs, and the database lncRNA2target to predict lncRNAs.

### The cancer genome atlas (TCGA) database analysis

To verify the results of the overlap and crosstalk networks, we used TCGA database (https://cancergenome.nih.gov) to analysis the expression of target genes and their effect on the survival rate of CRC patients. Cbioportal (http://www.cbioportal.org) was used to analysis 276 CRC samples in TCGA database. Enter the information to the web page of Cbioportal as following: 1) Select Genomic Profiles: mRNA Expression data; 2) Select Patient/Case Set: Tumors with sequencing and CAN data (212); 3) Enter Gene Set: the target genes; 4) Click on “OncoPrint” and “Survival”. Logrank test was used to analysis the significance of survival rate. Finally, we used Illustrator software to combine the survival rate files.

## Results

### Identification of DEGs in PMCT and LMCT

**Fig 1:**
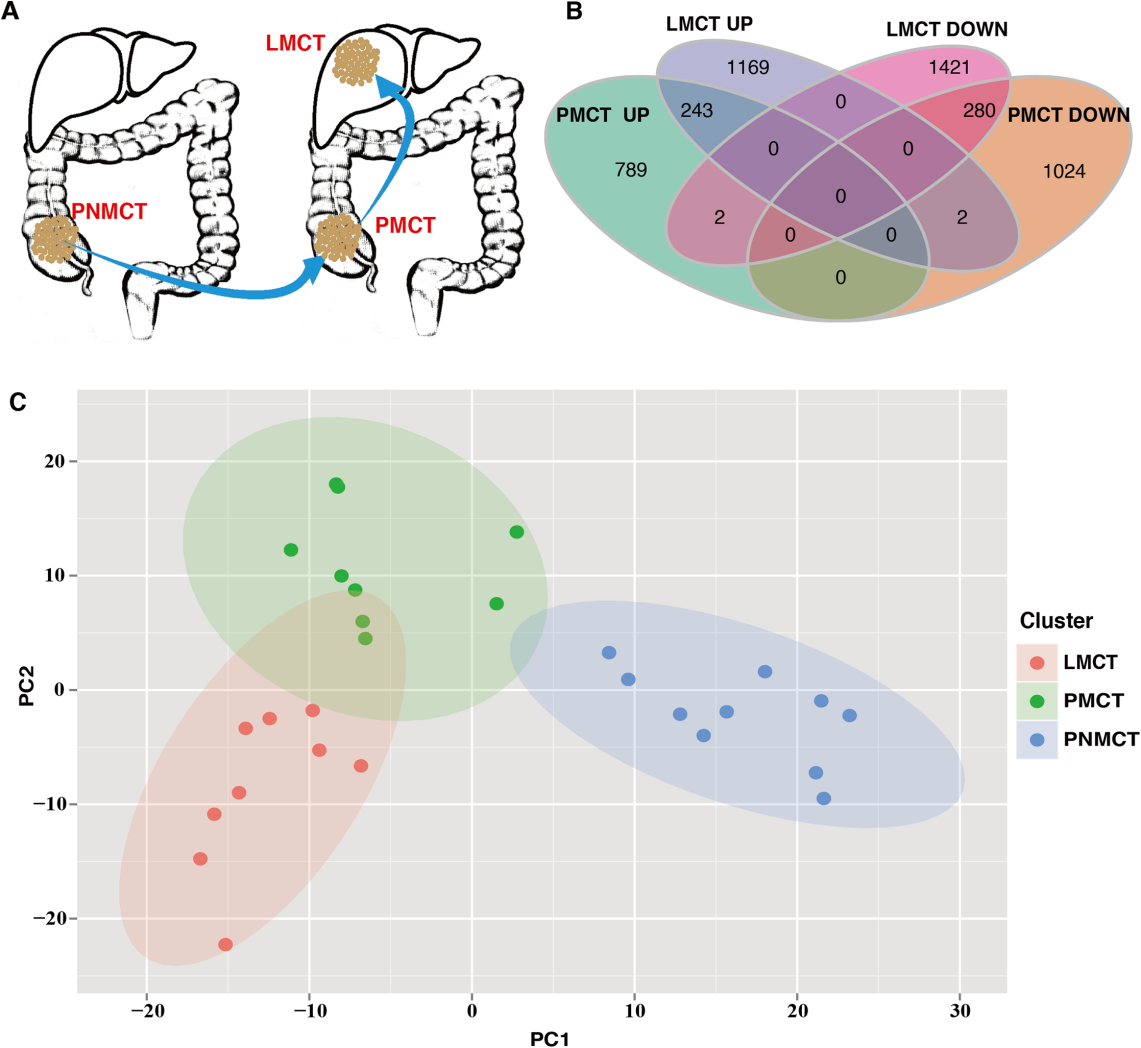
Sequencing data analysis. (A) Illustration of the 3 groups of tumor samples (i.e., PNMCT, PMCT and LMCT) that have been sequenced. (B) Venn diagram of DEGs. UP and DOWN represent up-regulated and down-regulated genes, respectivity. (C) Validation of the correlation between DEGs and samples. LMCT, PMCT and PNMCT samples are represented as red, green and blue ellipses, respectivity.

Based on the dynamic process of tumor metastasis, we collected PNMCT samples from 10 patients with CRC who had no liver metastases within ten years of follow up, PMCT and LMCT samples from 9 patients with CRC who had liver metastases. (Fig 1A) We sequenced these samples and established a heatmap of DEGs. Analyzing the DEGs using the R limma package[16], we identified 2060 DEGs (1034 up-regulated; 1026 down-regulated) and 2837 DEGs (1414 up-regulated; 1423 down-regulated) in PMCT and LMCT, respectively. A Venn diagram shows that there are a total of 527 overlapping DEGs (243 up-regulated; 280 down-regulated) in the two groups. (Fig 1B)

### Validation of the correlation between the DEGs and the samples

To demonstrate the correlation between DEGs and the samples tested, we performed PCA on 28 samples using the expression matrix of all DEGs. The results show that 4370 DEGs divide the 28 samples into 3 groups, as follows: the PNMCT, PMCT, and LMCT groups (Fig 1C).

### Identification of PPI networks and functional modules in LMCT and PMCT

**Fig 2:**
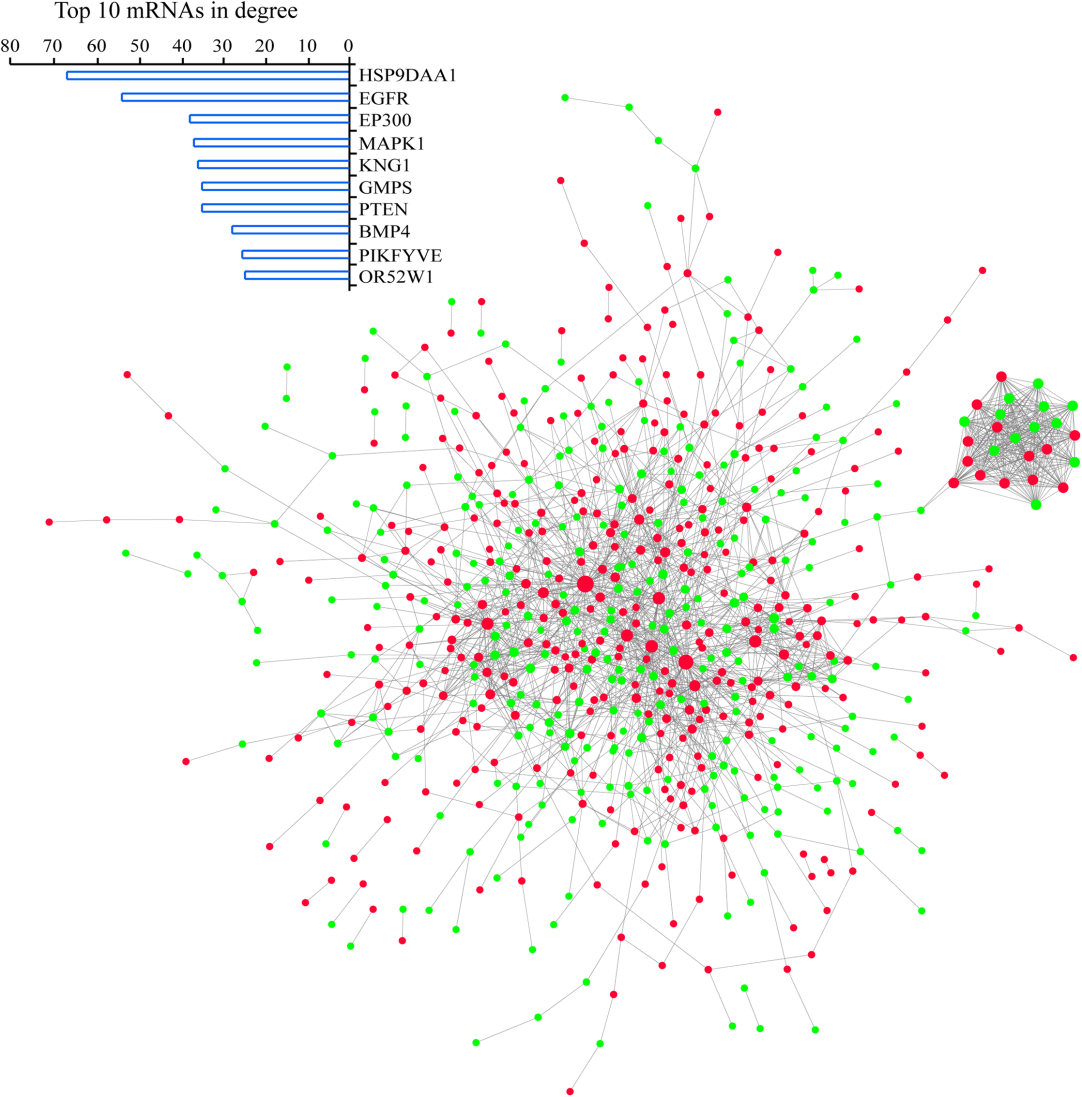
PPI network of PMCT. The STRING database was used to mine the PPI pairs of DEGs in PMCT. We identified 1836 PPI pairs among 489 differentially expressed genes and established a PPI network. Red and green nodes represent up-regulated and down-regulated genes, respectivety. The nodes represented by EGFR, EP300, MAPK1, KNG1, PTEN, and BMP4 in the PPI network had larger degree values.

To clarify the regulatory relationship of the DEGs between the LMCT and PMCT groups, we used the STRING database to mine the PPI pairs of DEGs in the two groups. We identified 1836 PPI pairs among 489 DEGs in the PMCT group and established a PPI network (Fig 2). The size of the nodes in this network indicates the degree value of the gene. The number of neighboring nodes directly connected to the node indicates the importance of the node in the network. The degree values of the network were then analyzed using Cytoscape software. The results show that gene nodes including EGFR, EP300, MAPK1, KNG1, PTEN, and BMP4 in the PPI network have larger degree values. To understand the major functions of the PPI network, we used the DAVID database (https://david.ncifcrf.gov) to perform a Gene Ontology (GO) analysis on the gene nodes in the PPI network. The results show that GO terms with a p value cutoff of 0.01 were primarily involved in the “positive regulation of cell proliferation” and “mitochondrial transport” (S1 Table). These findings are consistent with the massive proliferation of tumors cells in the primary lesions, which is one of the steps in the process of metastasis [4]. Furthermore, mitochondria are the most important energy-providing organelles in eukaryotic cells, and functional changes in mitochondria are closely associated with the progression and metastasis of tumors[17]. When we compared the PMCT group with the PNMCT group, we found that the genes in the PMCT group are consistent with increased proliferation and more active mitochondria. These results support the notion that cellular proliferation and increased energy reserves of individual tumor cells are associated with the capacity to metastasize.

**Fig 3:**
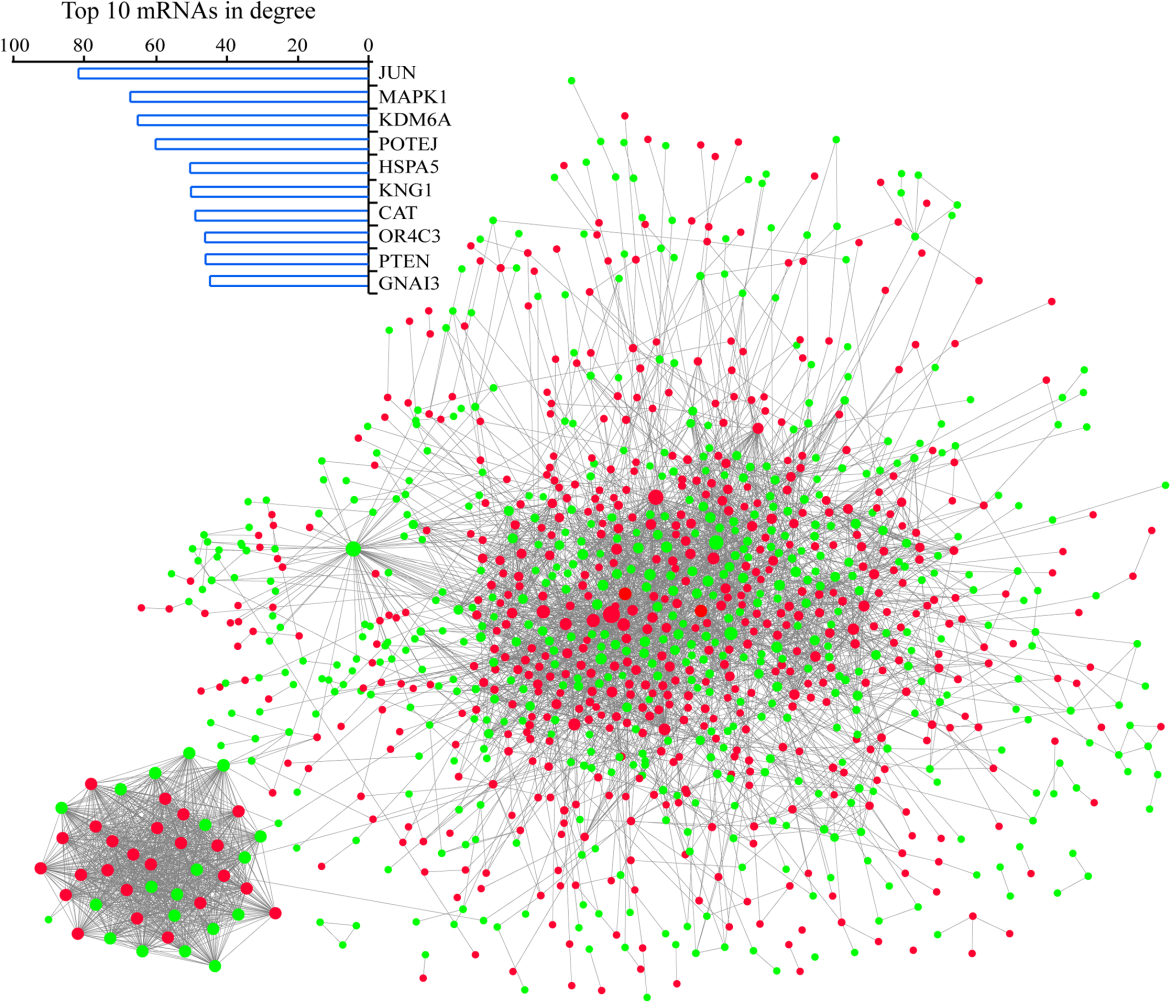
PPI network of LMCT. Using the STRING database, we identified 4729 PPI pairs among 1136 DEGs of LMCT and established a PPI network. Red and green nodes represent up-regulated and down-regulated genes, respectivety. The nodes represented by JUN, MAPK1, KDM6A, POTEJ, HSPA5, and KNG1 in the PPI network had larger degree values.

We identified 4729 PPI pairs among 1136 differentially expressed genes of LMCT and established a PPI network (Fig 3). The gene nodes JUN, MAPK1, KDM6A, POTEJ, HSPA5, and KNG1 in the PPI network have larger degree values. GO terms with a p value cutoff of 0.01 for the nodes in the PPI network include the positive regulation of metabolism of a variety of substances (e.g., “the positive regulation of macromolecules, nitrogen compounds, and RNA/nucleic acids”; S2 Table). Previous studies have shown that active carbohydrate and nucleic acid metabolism are features of the distant metastasis of tumors[18, 19]. Glucose metabolism can provide a large amount of energy to support tumor metastasis and can provide the raw materials for nucleic acid synthesis. The increase in nucleic acid metabolism suggests that the processes of transcription and translation of genes and the transmission of genetic information during tumor metastasis are more active. These results indicate that energy metabolism and cellular activity are increased in the PMCT group.

Using the PPI network of PMCT and LMCT, we identified the functional modules with the Cytoscape plugin MCODE. We identified 16 and 38 modules with an MCODE score cutoff of 1.5 in the PPI networks of PMCT and LMCT, respectively (S3 and S4 Tables).

### Modules with significant overlap between PMCT and LMCT

**Fig 4:**
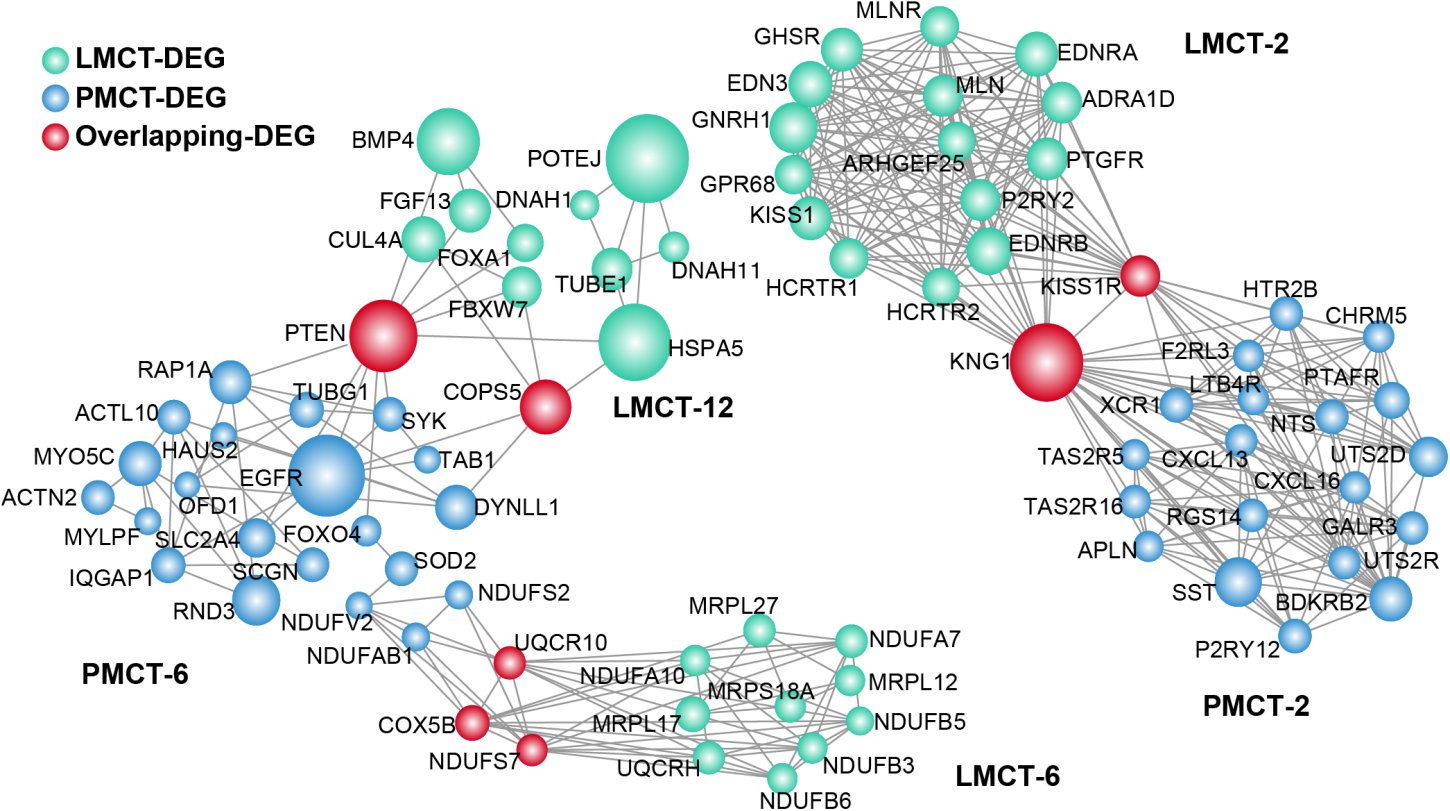
Modules with significant overlap between PMCT and LMCT. Each module was extracted after mapping DEGs to the human PPI network using the Cytoscape MCODE plugin. The module pairs with significant overlap between PMCT and LMCT were determined by a hypergeometric test with a cutoff of 0.05. Node size is shown according to its network degree. The module number is marked beside the module.

After performing the hypergeometric tests, three pairs of gene modules were found to have significant overlap between PMCT and LMCT with a p value cutoff of 0.05 (S5 Table). These 3 pairs of modules have a total of 82 DEGs including 7 overlapping DEGs (Fig 4).

PMCT-2 and LMCT-2 module pairs demonstrated overlaps in KNG1 and KISS1R. KNG1 is a potential prognostic marker of CRC, as the survival rate of CRC patients with positive KNG1 expression is lower than that of CRC patients with negative KNG1 expression[20]. In addition, Ji et al have shown that silencing KISS1 and KISS1R promotes the growth and metastasis of CRC cell lines *in vitro* suggesting that KISS1 and KISS1R are also potential therapeutic targets[21]. We found that the expression of KISS1 is decreased in PMCT and LMCT, which might result in the occurrence of CRC metastasis. The results of the GO analysis show that these 2 modules are both closely associated with G-protein coupled receptor (GPCR) signaling, which is also the GO term with the most statistical significance (p = 3.46E-11) (S6 Table). Liu et al have shown that GPCRs promote tumor metastasis in two ways: 1) they activate the Rho GTPases and change the cytoskeleton of cancer cells; 2) they provide nutrients for angiogenesis[22]. Therefore, GPCRs may also be crucial regulators for CRC liver metastasis.

The PMCT-6 and LMCT-6 module pairs demonstrated overlaps in COX5B, UQCR10, and NDUFS7. Wu et al have shown that COX5B is an important target of gastrin and that COX5B could regulate ATP metabolism and cell growth of CRC cells[23]. In addition, COX5B is also associated with tumor proliferation. Gao et al have shown that breast cancer cells exhibit proliferation inhibition and aging after COX5B knockout[24]. UQCR10 and NDUFS7 are both associated with mitochondrial function and cellular energy metabolism; however, their specific function in tumors remains unclear. GO analysis reveals that the functions of this module pair are all related to energy metabolism and “oxidative reduction”. Tumor proliferation and metastasis require a large amount of energy and tumor cells are provided with energy from a variety of sources through a series of oxidative reduction reactions[25]. Our results suggest that this group of overlapping modules might provide the energy for the development of CRC liver metastasis.

The PMCT-6 and LMCT-12 module pairs demonstrated an overlap in the PTEN and COPS5 genes. These 2 genes are both closely associated with CRC metastasis. Zhong et al have shown that COPS5 silencing significantly inhibits the ability of CRC cells to invade and it promotes cell cycle arrest[26]. Chowdhury et al have shown that the restoration of PTEN activity reduces liver metastasis of *in situ* CRC[27]. GO analysis shows that the functions of genes within this module are mainly associated with the formation of microtubule structure (i.e., “microtubule-based process”). As an important cytoskeletal component, microtubules maintain cell morphology, participate in cell movement and transport of intracellular materials, and are closely associated with tumor metastasis[28]. Our results indicate that both of these overlapping modules have functions related to the regulation of liver metastasis of CRC.

### Modules with significant crosstalk between PMCT and LMCT

**Fig 5:**
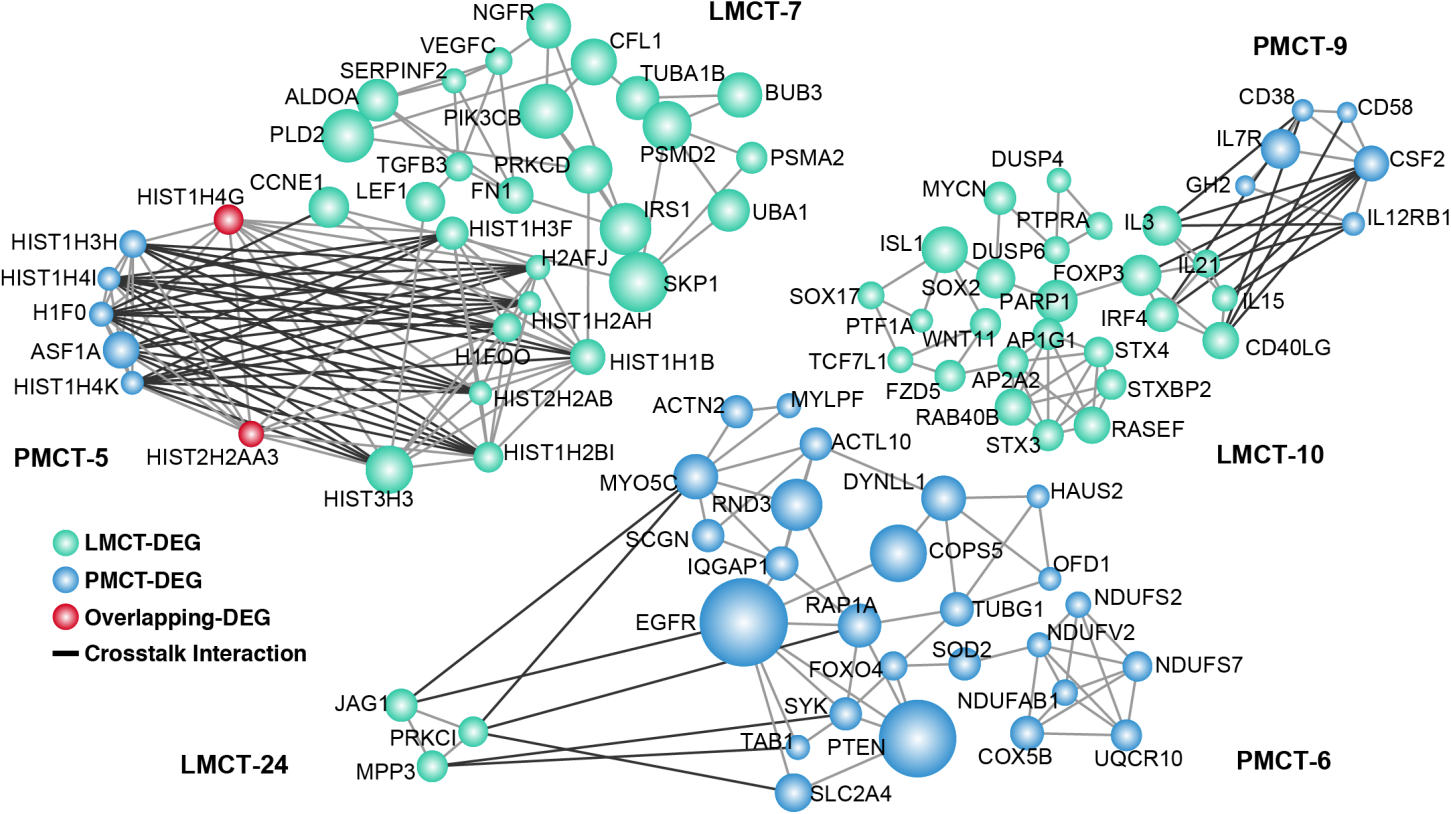
Modules with significant crosstalk between PMCT and LMCT. The module pairs with significant crosstalk were computed in comparison with 1000 random networks, with a p value cutoff of 0.05. Crosstalk interactions are shown in black. Node size is shown according to its network degree.

In addition to overlapping modules, the interaction among genes in different modules also featured crosstalk. After random computation and comparison, we found that the 3 pairs of PPI sub-networks between PMCT and LMCT had significant crosstalk with a p value cutoff of 0.05 (S7 Table). These 3 pairs of modules have a total of 95 significant DEGs including 2 overlapping DEGs, 56 DEGs in PMCT, 27 DEGs in LMCT, and 56 crosstalk pairs (Fig 5). GO analysis shows that the functions of genes in PMCT-5 and LMCT-7 are similar and are mainly involved in DNA metabolic processes such as DNA synthesis and assembly and cell cycle regulation. This increase in DNA synthesis suggests that cellular processes such as cell division and cell cycle regulation during tumor metastasis are even more active than in non-metastatic cells. Some studies have indicated that cell cycle-related genes are closely associated with tumor metastasis. For example, cyclin E1 (CCNE1) in the LMCT-7 module has been shown to promote metastasis of ovarian cancer and bladder cancer[29, 30]. The gene nodes in PMCT-5 (e.g., H1F0) might influence the expression and function of important genes (e.g., CCNE1) in the LMCT-7 module through crosstalk, which in turn regulates the cell cycle and metastasis of CRC cells. Therefore, this pair of modules with crosstalk interactions might be a bridge that connects PMCT and LMCT through the biological functions described above.

In the LMCT-10 and PMCT-9 module pairs, the functions of LMCT-10 are primarily related to immune regulation while the functions of PMCT-9 involve the positive regulation of cell proliferation. Tumor metastasis is closely associated with immune regulation in tumors. The immune escape mechanism helps circulating tumor cells to avoid immune-mediated cell death and facilitates the metastasis of tumor cells to distant organs[31]. It is worth noting that the genes that exhibit crosstalk in this pair of modules are mainly interleukins and their receptors (e.g., IL-3, IL-7R). Yu et al have shown that IL-3 promotes the growth and invasiveness of prostate cancer cells[32]. IL-7R is highly expressed in lung cancer and CRC and it promotes lung cancer vascular endothelial growth and metastasis[33]. Therefore, it is possible that these metastasis-related interleukins and receptors might promote metastasis of CRC and could serve as a bridge that connects the two disease modules.

Due to the combination of overlap and crosstalk within the energy metabolism-related module PMCT-6, this module might be the most important module in the 2 sub-networks. The PMCT-6 module not only overlapped with the 2 LMCT modules (LMCT-6 and LMCT-12) but also demonstrated crosstalk with LMCT-7 (S8 Table). Studies have shown that the 3 genes in LMCT-7 are all associated with tumor metastasis: PRKCI is over-expressed in lymph node metastasis of esophageal cancer and can be used as a biomarker of metastasis[34], JAG1 can promote metastasis of breast cancer and lung cancer[35, 36], and MPP3 can promote the migration and invasiveness of liver cancer[37]. This LMCT-7 module might have the same function (i.e., the promotion of tumor metastasis) in CRC. Our results also show that EGFR is an important gene node in PMCT-6. EGFR is one of the most important receptors of tumor cell growth via a variety of signaling pathways. Some studies have shown that the expression of the metastasis-promoting gene JAG1 in lung cancer cells depends on activation by EGFR[38]. Our results also reveal that the EGFR and JAG1 crosstalk pair are expressed during the process of liver metastasis in cases of CRC. Therefore, EGFR in PMCT might promote CRC liver metastasis through the alteration of JAG1 expression in LMCT.

### Transcription regulation networks of modules with significant overlap and crosstalk between PMCT and LMCT

**Fig 6:**
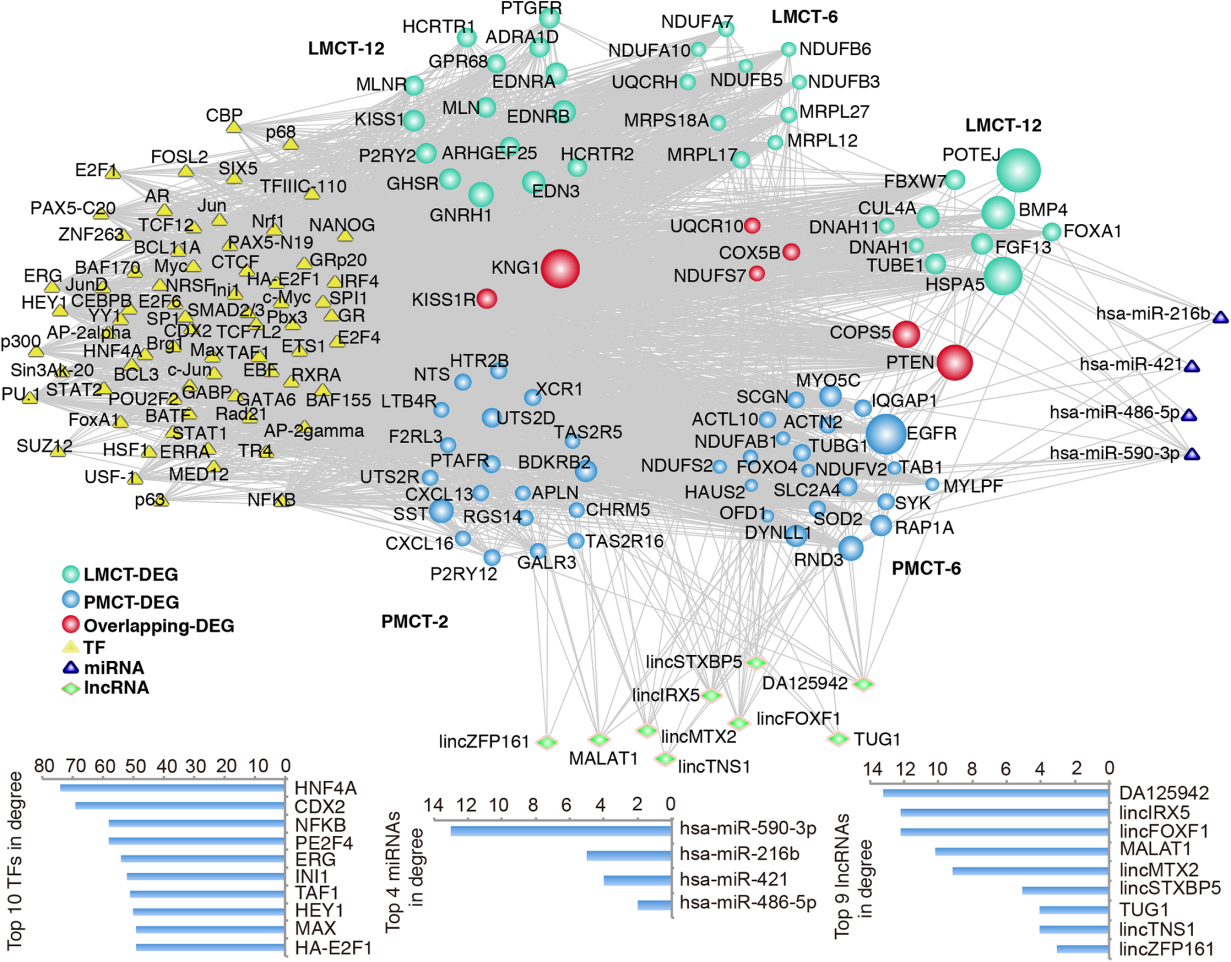
Transcription regulation networks of modules with significant overlap between PMCT and LMCT. TFs and miRNAs were computed based on the number of their interactions with the module pair and the enrichment significance of their regulating targets. lncRNAs were predicted using the lncRNA2target database. Network nodes are colored as PMCT, LMCT DEGs and overlapping DEGs with size showing their network degree. TFs are represented as white triangles, miRNAs are grey triangles, while lncRNAs are yellow diamonds.

**Fig 7:**
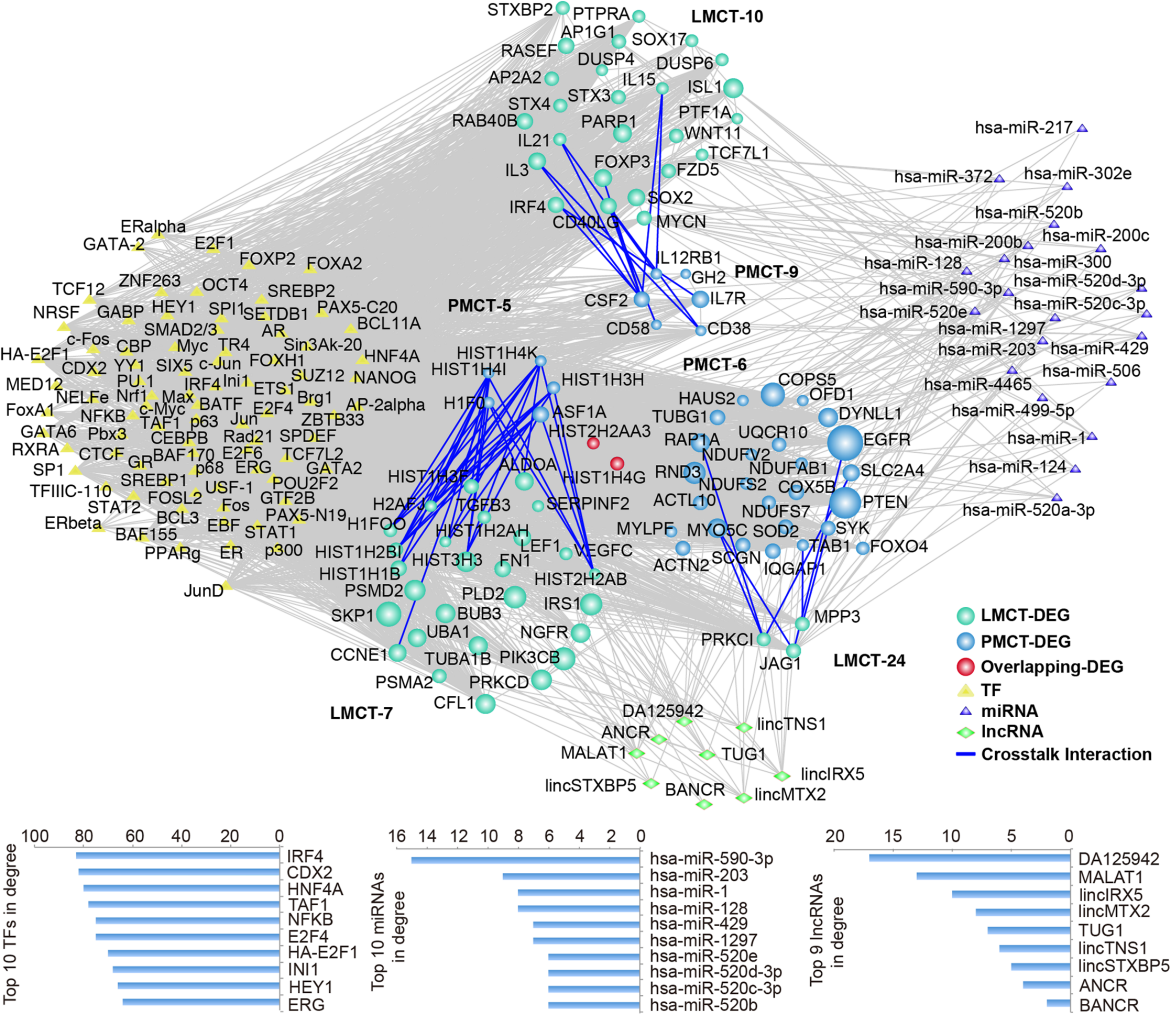
Transcription regulation networks of modules with significant crosstalk between PMCT and LMCT. Network nodes are colored as PMCT and LMCT DEGs with their size showing their network degree. TFs are represented as white triangles, pivot miRNAs are grey triangles, while lncRNAs are yellow diamonds.

To clarify the underlying transcription and post-transcription regulation networks of the modules with overlap and crosstalk, we explored the miRNAs, lncRNAs, and TFs that are responsible for the regulation of genes in these 2 modules. A total of 4 upstream miRNAs, 9 upstream lncRNAs, and 69 TFs that met the conditions were predicted among genes in the 82 overlapping network modules (Fig 6). DEGs in the 9 network modules with significant crosstalk were used to predict miRNAs, lncRNAs, and TFs, and the results showed that 24 upstream miRNAs, 9 lncRNAs, and 84 TFs met the conditions (p<0.05) (Fig 7).

We found that some of the TFs that we identified have already been implicated in the regulation of liver metastasis in the setting of CRC. For example, CDX2 is a TF with tumor suppressor functions that has been shown to be minimally expressed in CRC cell lines; the over-expression of CDX2 significantly inhibits the invasive ability of CRC cells[39]. Pancione et al have reported that dysregulation of INI1 could promote the occurrence of CRC liver metastasis [40]. Lwamoto et al have reported that the over-expression of E2F1 could promote metastases of CRC to the liver and lung[41]. These results, to some extent, validate the accuracy of our predicted transcription regulation networks. In addition, we identified some TFs that function in the regulation of tumor metastasis. For example, HNF4A, which can promote the metastasis of liver cancer, was shown to be closely associated with the epithelial-mesenchymal transition (EMT)[42]; E2F4 has been shown to be minimally expressed in breast cancer and is associated with the occurrence of metastasis[43]; and ERG has been shown to promote metastasis of prostate cancer[44]. The roles of these TFs in the regulation of metastasis of CRC have not been studied; however, these TFs could be potential targets for the treatment of CRC liver metastasis. Notably, among the top 10 TFs, 9 TFs including CDX2 and INI1 could regulate both the overlapping and crosstalk networks, which suggests that they might have broader functions than the TFs that only regulate a single network.

In addition to TFs, non-coding genes are also very important for the post-transcriptional regulation of overlapping and crosstalk networks. In the transcription regulation networks of overlapping modules, all of the miRNAs that we identified are closely associated with tumor metastasis. For example, miR-216b and miR-486-5p inhibit invasion and metastasis of liver cancer cells[45, 46], while miR-421 can promote metastasis of gastric cancer and neuroblastoma[47]. In the transcription regulation networks of crosstalk modules, miR-1[48], miR-128[49], miR-1297 [50], and miR-429[51] all have inhibitory functions on the metastasis of CRC, which further validates the accuracy of our prediction. Furthermore, some of other miRNAs we identified are also closely associated with metastasis of other tumor types. For example, miR-203 can inhibit the proliferation and metastasis of liver cancer[52], miR-520d-3p can inhibit the proliferation and metastasis of gastric cancer[53], and miR-520b can inhibit the migration of breast cancer cells[54]. miR-590-3p had the largest degree value in the two networks when the results were combined for these two networks. Pang et al have reported that miR-590-3p can inhibit the migration, invasion, and EMT of glioblastoma[55]. Our miRNA prediction results suggest that besides the 4 miRNAs that have been demonstrated to regulate the metastatic capacity of CRC, other miRNAs also have the ability to regulate tumor metastasis through the regulation of genes in modules that demonstrate overlap and crosstalk. Therefore, these miRNAs represent potential novel targets for the treatment of liver metastasis in patients with CRC.

In contrast to miRNAs that have been extensively investigated, studies on the functions of lncRNAs are still at the initial stage, and the functions of many lncRNAs are still unclear. However, recent studies have indicated that some of our screened lncRNAs possess regulatory functions in the metastasis of CRC. For example, MALATA1 and BANCR can promote the migration and invasion of CRC[56, 57], while TUG1, which is associated with a poor prognosis, can promote the development of EMT and the metastasis of CRC cells[58]. Although some lncRNAs have not been studied in CRC liver metastasis, lncRNAs have the ability to regulate the metastasis of other types of tumors. For example, lincIRX5 can promote the invasion and metastasis of gliomas[59]. Moreover, lincFOXF1 can inhibit the metastasis of gastric cancer cells[60]. The functions of other lncRNAs have not yet been elucidated. lncRNAs with a demonstrated ability to regulate tumor metastasis (e.g., lincIRX5 and lincFOXF1) and lncRNAs with unclear functions (e.g., lincMTX2 and lincTNS1) might have the potential to regulate CRC liver metastasis in a manner similar to that of TUG1 and MALAT1.

### TCGA database analysis

**Fig 8:**
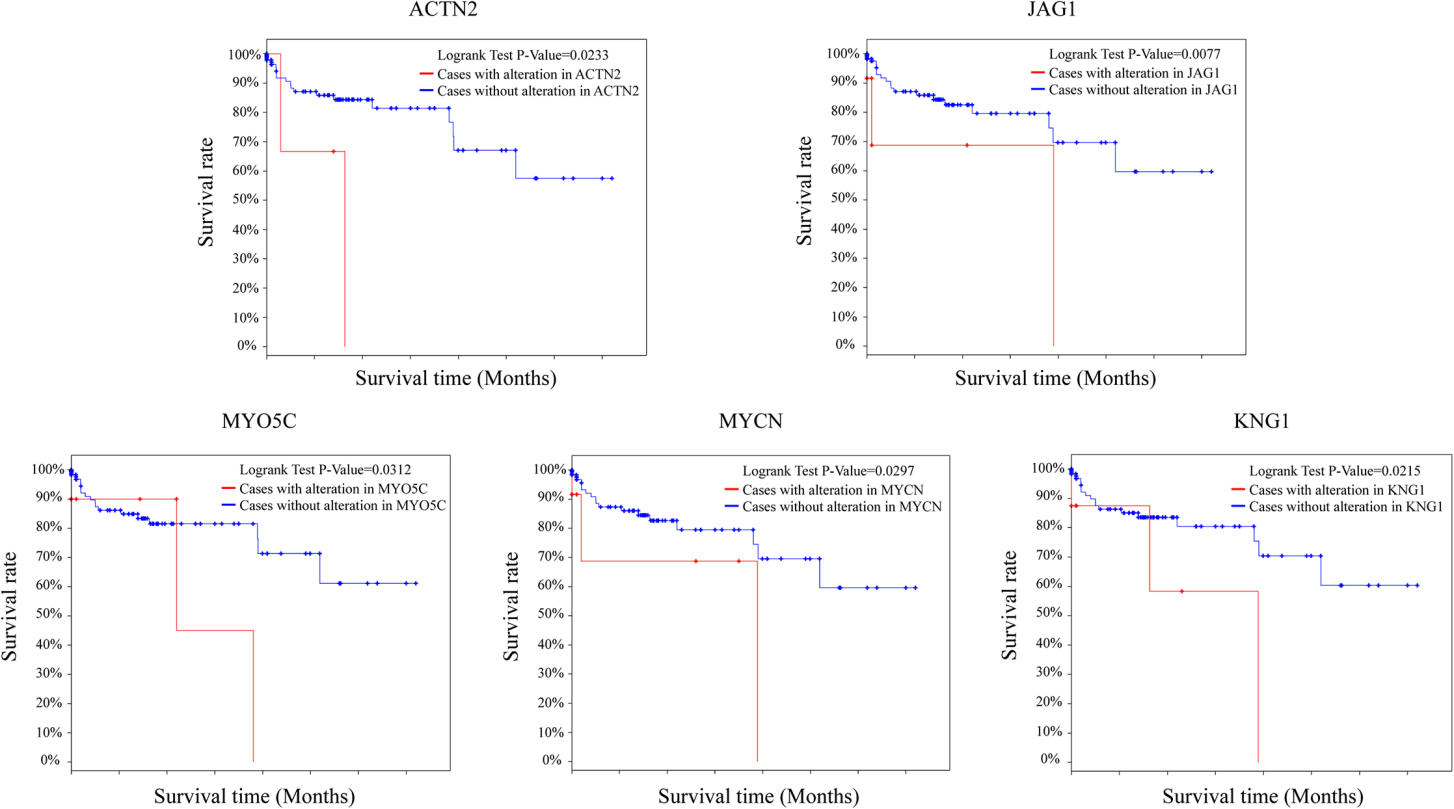
The effect of the target genes on the survival rate of CRC patients in TCGA database. Among 276 CRC samples in TCGA database, we found the CRC patients with alteration in two overlap network genes KNG1 (p = 0.0215) or MYO5C (p = 0.0312) had lower survival rates than the CRC patients with normal KNG1 or MYO5C expression. Moreover, the CRC patients with alteration in three crosstalk network genes JAG1 (p = 0.0077) or ACTN2 (p = 0.0233) or MYCN (p = 0.0297) had lower survival rates than the CRC patients with normal JAG1 or ACTN2 or MYCN expression.

We used TCGA database to analysis the expression of target genes in overlap and crosstalk network and their effect on the survival rate of CRC patients. The results showed that five target genes in the overlap and crosstalk networks were differentially expressed in CRC samples and could effect the survival rate of CRC patients. (i.e., JAG1, KNG1, MYO5C, MYCN and ACTN2) Among 276 CRC samples in TCGA database, we found the CRC patients with alteration in KNG1 (p = 0.0215) or MYO5C (p = 0.0312) had lower survival rates than the CRC patients with normal KNG1 or MYO5C expression. Moreover, the CRC patients with alteration in JAG1 (p = 0.0077) or ACTN2 (p = 0.0233) or MYCN (p = 0.0297) had lower survival rates than the CRC patients with normal JAG1 or ACTN2 or MYCN expression. (Fig 8) In this study, we used a multidimensional integration analysis to establish the overlap and crosstalk network in the process of CRC liver metastasis. We identified KNG1 and MYO5C in the overlap network and MYCN, ACTN2 and JAG1 in the crosstalk network. The results of TCGA analysis validated the results of sequencing and screening in this study.

## Discussion

High-throughput next generation sequencing offers a powerful tool for studying the underlying genetic alterations of a disease process. Using this technology, we studied the genetic changes that underlie different stages (from PNMCT through PMCT to LMCT) of CRC liver metastasis. To fully unveil the global perspective provided by high-throughput sequencing data, we applied an integration methodology based on sequencing data, PPI networks, transcription regulation, and some non-coding RNA prediction databases to discover bridging targets of CRC from a non-metastatic state to a metastatic state.

Our established overlapping networks revealed 7 overlapping genes between the related modules of PMCT and LMCT. These genes might regulate a variety of biological processes associated with tumor metastasis including the regulation of GPCR signaling, cell proliferation, and energy metabolism. As demonstrated, some overlapping genes such as KISS1R, COPS5, and PTEN have been shown to have important regulatory functions in the process of CRC metastasis [21, 26, 27]. Interestingly, modules that overlap usually have similar biological functions. For example, the PMCT-6 and LMCT-6 module pairs are both associated with energy metabolism while the PMCT-6 and LMCT-12 module pairs are both associated with “microtubule-based processes”. These results reveal a possible mechanism of CRC liver metastasis: metastasis-related gene modules in PMCT could use the bridging function of overlapping genes to induce changes in metastasis-related gene modules in LMCT. Furthermore, these modules usually have a similar metastasis-related function, which suggests that they might also participate in the transmission and enhancement of metastasis-related functions in the process of tumor development. In addition to their overlapping relationship, the PMCT and LMCT modules display interactive functions through crosstalk. GO analysis shows that the functions of these modules with crosstalk are all closely associated with tumor activities. For example, the LMCT-7 module can regulate the cell cycle, the PMCT-9 module can regulate cell proliferation, and the LMCT-10 module can regulate immune responses. Our findings suggest that PMCT modules might influence LMCT modules through the occurrence of crosstalk. For example, interleukin receptors (IL-17R and IL-21RB1) in PMCT-9 can influence the action of interleukin genes (IL-3, -15 and -21) in LMCT-10 through crosstalk. Studies have shown that interleukins and their receptors are closely associated with tumor metastasis; for example, IL-3 and IL-7R in different modules can promote both the proliferation and metastasis of tumors[32, 33]. The combination of overlap and crosstalk among module networks (Figs 4 and 5) suggests that PMCT-6 might be a key module associated with CRC metastasis. PMCT-6 not only influences the LMCT module through overlap to regulate energy metabolism in CRC liver metastasis, but it also influences LMCT-7 through crosstalk, which then promotes CRC metastasis to the liver.

Based on a comprehensive strategy integrating many types of databases and statistical algorithms, we constructed transcription regulation networks including TFs, miRNAs, and lncRNAs between the PMCT and LMCT groups, each of which had modules with considerable overlap and crosstalk. After these 2 networks were combined, we found that 9 of the top 10 TFs could regulate gene nodes in these 2 networks. Some of these genes, such as CDX2, INI1, and E2F1, have been shown to influence the metastasis of CRC[39–41], which validates the accuracy of our screening. We have also found other TFs that have not previously been shown to be associated with CRC liver metastasis. Analysis of the screened miRNAs shows that miRNAs in the overlapping network are all associated with tumor metastasis; however, none of them have been studied in CRC liver metastasis. Some miRNAs are able to inhibit CRC liver metastasis through the regulation of networks with significant crosstalk, such as miR-1, miR-128, miR-1297, and miR-429[48–51]. Although miR-520d-3p, miR-203 and miR-590-3p have been reported to inhibit other types of tumor metastasis, no functional studies have been conducted on their role in CRC liver metastasis. Based on these results, we propose that these miRNAs could potentially serve as novel targets for the treatment of CRC liver metastasis. Furthermore, we also screened lncRNAs that could regulate the two networks. Similar to our results with the TFs, 7 of the top 9 lncRNAs could regulate the two networks. Of these, MALTA1 and TUG1 have been shown to promote the occurrence of the EMT, invasion, and metastasis of CRC cells[56, 58]. Although no reports have been published on the role of lincIRX5 and lincFOXF1 in CRC liver metastasis, these genes have been shown to regulate metastasis of other types of tumors. Thus far, only a few relevant studies have been published on lncRNAs and CRC liver metastasis. Our predicted lncRNAs might serve as a basis for new studies on the mechanism of CRC liver metastasis.

In summary, using next generation sequencing data from tumor samples, we used an integrated approach to explore the potential molecular targets that play a bridging role in the process of CRC liver metastasis. Our results demonstrate the power of a global and multidimensional systematic approach on the discovery of potential molecular mechanisms of tumor metastasis. This strategy not only unveils the dynamic links between PNMCT, PMCT and LMCT, but could also be applicable to the study of other tumor metastasis, such as breast cancer with bone metastasis, hepatocellular carcinoma with lung metastasis and lung cancer with brain metastasis. In addition to further study on validated molecules, more efforts should be made to study the remaining potential molecular targets (e.g., KNG1, E2F4, miR-590-3p and lincIRX5). These new molecular targets may also play an important role in CRC liver metastasis and could be new tools for research on mechanisms in the future.

## Supporting information

S1 Table The function of node genes in PMCT PPI network. (DOCX)

S2 Table The function of node genes in LMCT PPI network. (DOCX)

S3 Table Gene modules in PMCT. (DOCX)

S4 Table Gene modules in LMCT. (DOCX)

S5 Table Modules with significant overlap. (DOCX)

S6 Table The function of modules with significant overlap. (DOCX)

S7 Table Modules with significant crosstalk. (DOCX)

S8 Table The function of modules with significant crosstalk. (DOCX)
