# Supplementary material for: A multidimensional integration analysis reveals potential bridging targets in the process of colorectal cancer liver metastasis
Source: PLoS One. 2017 Jun 19;12(6):e0178760. doi: 10.1371/journal.pone.0178760 (PMC5476238; doi:10.1371/journal.pone.0178760)
Supplement: S1 Table — (DOCX) [file pone.0178760.s001.docx]

| GO Term | P Value |
| --- | --- |
| GO:0042127~regulation of cell proliferation | 8.43E-04 |
| GO:0050730~regulation of peptidyl-tyrosine phosphorylation | 0.00493 |
| GO:0006839~mitochondrial transport | 0.00539 |
| GO:0008284~positive regulation of cell proliferation | 0.006633 |
| GO:0051247~positive regulation of protein metabolic process | 0.007134 |
| GO:0050877~neurological system process | 0.007968 |
| GO:0002703~regulation of leukocyte mediated immunity | 0.00953 |

**Supplemental Table 1: The function of node genes in PMCT PPI network**
