# Supplementary material for: A multidimensional integration analysis reveals potential bridging targets in the process of colorectal cancer liver metastasis
Source: PLoS One. 2017 Jun 19;12(6):e0178760. doi: 10.1371/journal.pone.0178760 (PMC5476238; doi:10.1371/journal.pone.0178760)
Supplement: S2 Table — (DOCX) [file pone.0178760.s002.docx]

**Supplemental Table 2: The function of node genes in LMCT PPI network**

| GO Term | P Value |
| --- | --- |
| GO:0001763~morphogenesis of a branching structure | 2.47E-04 |
| GO:0010604~positive regulation of macromolecule metabolic process | 8.37E-04 |
| GO:0048754~branching morphogenesis of a tube | 8.46E-04 |
| GO:0007608~sensory perception of smell | 0.001033 |
| GO:0051254~positive regulation of RNA metabolic process | 0.001073 |
| GO:0010557~positive regulation of macromolecule biosynthetic process | 0.001546 |
| GO:0009891~positive regulation of biosynthetic process | 0.001717 |
| GO:0031328~positive regulation of cellular biosynthetic process | 0.001868 |
| GO:0032868~response to insulin stimulus | 0.001898 |
| GO:0045935~positive regulation of nucleobase, nucleoside, nucleotide and nucleic acid metabolic process | 0.002141 |
| GO:0007242~intracellular signaling cascade | 0.002505 |
| GO:0045893~positive regulation of transcription, DNA-dependent | 0.00261 |
| GO:0051173~positive regulation of nitrogen compound metabolic process | 0.002739 |
| GO:0002088~lens development in camera-type eye | 0.002836 |
| GO:0030855~epithelial cell differentiation | 0.003228 |
| GO:0060429~epithelium development | 0.003346 |
| GO:0035239~tube morphogenesis | 0.00341 |
| GO:0007600~sensory perception | 0.003596 |
| GO:0050877~neurological system process | 0.004438 |
| GO:0007606~sensory perception of chemical stimulus | 0.004559 |
| GO:0070201~regulation of establishment of protein localization | 0.004992 |
| GO:0065004~protein-DNA complex assembly | 0.005668 |
| GO:0050890~cognition | 0.005948 |
| GO:0045941~positive regulation of transcription | 0.006244 |
| GO:0060041~retina development in camera-type eye | 0.006385 |
| GO:0051223~regulation of protein transport | 0.00674 |
| GO:0010628~positive regulation of gene expression | 0.007169 |
| GO:0006334~nucleosome assembly | 0.007701 |
| GO:0032880~regulation of protein localization | 0.007913 |
| GO:0006323~DNA packaging | 0.008535 |
| GO:0048660~regulation of smooth muscle cell proliferation | 0.008737 |
| GO:0045944~positive regulation of transcription from RNA polymerase II promoter | 0.009919 |
