# Supplementary material for: A multidimensional integration analysis reveals potential bridging targets in the process of colorectal cancer liver metastasis
Source: PLoS One. 2017 Jun 19;12(6):e0178760. doi: 10.1371/journal.pone.0178760 (PMC5476238; doi:10.1371/journal.pone.0178760)
Supplement: S3 Table — (DOCX) [file pone.0178760.s003.docx]

**Supplemental Table 3: Gene modules in PMCT**

| Module number | Score | Nodes | Edges | Node Genes |
| --- | --- | --- | --- | --- |
| 1 | 23.077 | 25 | 300 | OR4C5, OR4X1, OR51F1, OR9Q1, OR6J1, OR2T12, OR10A7, OR5C1, OR5J2, OR6K3, OR7C2, OR5P2, OR11L1, OR52W1, OR2B6, OR52K1, OR4K17, OR2A2, OR8B12, OR2A7, OR1L3, OR51E2, OR5K4, OR5L1, OR13H1 |
| 2 | 11.273 | 21 | 124 | PTAFR, CXCL16, GALR3, HTR2B, APLN, UTS2R, TAS2R5, RGS14, BDKRB2, SST, P2RY12, TAS2R16, NTS, XCR1, KISS1R, CHRM5, UTS2D, F2RL3, KNG1, LTB4R, CXCL13 |
| 3 | 7.818 | 10 | 43 | RPL12, TPT1, MRPL22, MRPL1, RPL31, RPL39, RPL30, RPS9, MRPS2, RPL21 |
| 4 | 5.25 | 15 | 42 | PSMA2, PSME1, ODC1, EP300, RAD23B, PSMD10, PSMA7, MAPK1, HNF4A, CDC26, KLHL12, PSMD3, BMP4, UBQLN2, PSMA4 |
| 5 | 4.5 | 7 | 18 | H1F0, HIST1H3H, HIST1H4I, HIST2H2AA3, ASF1A, HIST1H4K, HIST1H4G |
| 6 | 3.926 | 26 | 53 | SCGN, TUBG1, COPS5, DYNLL1, PTEN, SOD2, RND3, MYO5C, MYLPF, NDUFAB1, NDUFS2, NDUFS7, RAP1A, ACTN2, HAUS2, FOXO4, ACTL10, NDUFV2, COX5B, UQCR10, IQGAP1, SLC2A4, SYK, TAB1, EGFR, OFD1 |
| 7 | 2.9 | 39 | 58 | NOC4L, OSM, KRT8, IL4, TDGF1, SOX2, HNRNPA0, PDE6G, GUCY1A3, NCL, HNRNPF, SNRPC, PDE6A, BAD, YWHAQ, MYL7, RXFP2, ALDOA, MMP14, PPP1R16A, PPP1CA, TIMP3, RPS6KA2, RNPC3, BOLL, GUCY1A2, GMPS, COL4A1, PDE4C, IRS1, MAK16, PGD, TKTL2, ACTL6A, PRDM14, SF3B5, CSF2RB, FOXD3, UTP15 |
| 8 | 2.75 | 15 | 22 | GRIN2D, ARID3A, WSB2, TTC24, GRIN3B, SETD1A, PPIF, HSBP1, PPIB, TIMM44, GML, GRID1, WDR5B, HSP90AA1, VDAC2 |
| 9 | 2.286 | 6 | 8 | CSF2, IL7R, CD38, GH2, IL12RB1, CD58 |
| 10 | 1.6 | 4 | 4 | KCNJ5, KCNK9, KCNN4, KCNE2 |
| 11 | 1.5 | 3 | 3 | GRPEL2, PIKFYVE, HSPA13 |
| 12 | 1.5 | 3 | 3 | KLHDC5, KLHL9, KLHL8 |
| 13 | 1.5 | 3 | 3 | FKTN, POMGNT1, FKRP |
| 14 | 1.5 | 3 | 3 | BLOC1S6, HPS4, BLOC1S2 |
| 15 | 1.5 | 3 | 3 | SPATA16, PICK1, DPY19L2 |
| 16 | 1.5 | 3 | 3 | ZNF597, THTPA, SPINK6 |
