# Supplementary material for: A multidimensional integration analysis reveals potential bridging targets in the process of colorectal cancer liver metastasis
Source: PLoS One. 2017 Jun 19;12(6):e0178760. doi: 10.1371/journal.pone.0178760 (PMC5476238; doi:10.1371/journal.pone.0178760)
Supplement: S4 Table — (DOCX) [file pone.0178760.s004.docx]

**Supplemental Table 4: Gene modules in LMCT**

| Module number | Score | Nodes | Edges | Node Genes |
| --- | --- | --- | --- | --- |
| 1 | 41 | 43 | 902 | OR4C5, OR3A2, OR52M1, OR10H1, OR4D6, OR10AG1, OR2AG2, OR5M11, OR5H6, OR2T2, OR7E24, OR1F1, OR10A7, OR6J1, OR4K2, OR4K15, OR2G3, OR4C3, OR10A2, OR1J1, OR2B6, OR5D18, OR51I2, OR4D5, OR8A1, OR6T1, OR4N2, OR4F5, OR4X1, OR4M1, OR2AJ1, OR10A4, OR8D2, OR5AS1, OR5L2, OR7C2, OR2A7, OR5M3, OR51F1, OR52A5, OR8B12, OR5L1, OR9Q2 |
| 2 | 15.111 | 17 | 136 | EDN3, GPR68, KISS1, HCRTR2, GNRH1, P2RY2, ADRA1D, MLN, ARHGEF25, GHSR, HCRTR1, MLNR, PTGFR, EDNRB, KISS1R, KNG1, EDNRA |
| 3 | 12.414 | 28 | 180 | RPS4Y2, MRPL13, DDX55, EIF3G, DDX56, RPS21, NCBP1, RPL22, SMG8, DDX28, BOP1, NIP7, EIF1AX, NHP2, DDX52, WDR12, RPL21, DHX37, PABPC1, RBM28, RPL3L, RPS4X, MRPS2, MAGOH, KCTD19, DIEXF, RPLP1, RPL30 |
| 4 | 10.154 | 12 | 66 | GNAI3, SST, RXFP4, OPRL1, C5, P2RY4, OPRK1, MTNR1B, FPR1, GNAI1, HTR1D, CXCR5 |
| 5 | 7.429 | 20 | 78 | RORB, IFNA7, IFNA16, IFNB1, IL15RA, MAP2K1, IL7R, IFNG, NR2F6, ESR2, HNF4G, IL20RA, NR0B2, NR2E1, NR4A2, IFNA5, MAPK1, NR1D2, TYK2, JAK1 |
| 6 | 6.143 | 13 | 43 | NDUFA7, UQCRH, NDUFB6, MRPL17, NDUFS7, UQCR10, NDUFA10, NDUFB5, COX5B, MRPS18A, NDUFB3, MRPL27, MRPL12 |
| 7 | 4.8 | 29 | 72 | HIST2H2AA3, UBA1, H1FOO, ALDOA, PRKCD, TGFB3, HIST1H1B, SERPINF2, FN1, PLD2, HIST3H3, PSMD2, PIK3CB, H2AFJ, HIST2H2AB, CFL1, CCNE1, HIST1H2BI, IRS1, HIST1H3F, TUBA1B, BUB3, VEGFC, PSMA2, SKP1, LEF1, HIST1H4G, HIST1H2AH, NGFR |
| 8 | 4.5 | 11 | 27 | PAK2, PSMA4, RASL12, PSMA5, PSMD10, UBE2E1, RRAD, SKP2, ITSN2, RND3, RHOH |
| 9 | 3.765 | 16 | 32 | ARF4, RAB7A, TSG101, ATXN2, ATXN3, CAT, ATXN2L, VPS8, VPS45, JUN, ICAM1, VPS39, ELANE, SNX1, EEA1, RAB22A |
| 10 | 3.385 | 25 | 44 | IL15, CD40LG, SOX17, IL21, FZD5, IRF4, STX3, TCF7L1, ISL1, RASEF, DUSP4, PTPRA, STXBP2, IL3, WNT11, STX4, SOX2, RAB40B, DUSP6, MYCN, FOXP3, PTF1A, PARP1, AP1G1, AP2A2 |
| 11 | 3.333 | 5 | 10 | GABRA2, GABRR2, FXYD3, CLCN1, GABRB1 |
| 12 | 2.769 | 12 | 18 | CUL4A, BMP4, POTEJ, DNAH11, COPS5, FOXA1, DNAH1, TUBE1, FGF13, HSPA5, PTEN, FBXW7 |
| 13 | 2.571 | 6 | 9 | TK1, CASP7, CASP6, BCL2L14, SP1, TFDP1 |
| 14 | 2.4 | 4 | 6 | DHX38, SNRPD1, SF3B5, PTBP1 |
| 15 | 2.4 | 4 | 6 | CLDN15, CLDN5, CLDN17, CLDN22 |
| 16 | 2 | 4 | 5 | ACTR1A, BBS10, ACTR1B, DNHD1 |
| 17 | 2 | 4 | 5 | CDS2, GPAT2, AGPAT1, AGPAT9 |
| 18 | 2 | 4 | 5 | LGALS7B, FUT1, FUT8, ST3GAL1 |
| 19 | 2 | 4 | 5 | ABLIM2, LCE3E, ADNP2, LCE5A |
| 20 | 1.5 | 3 | 3 | PDHA1, PDHA2, DLAT |
| 21 | 1.5 | 3 | 3 | EPC1, MORF4L2, MRGBP |
| 22 | 1.5 | 3 | 3 | NBPF3, ZNF543, RNASE7 |
| 23 | 1.5 | 3 | 3 | CTH, AHCYL2, BHMT |
| 24 | 1.5 | 3 | 3 | JAG1, PRKCI, MPP3 |
| 25 | 1.5 | 3 | 3 | FOXA2, PCK1, PFKFB1 |
| 26 | 1.5 | 3 | 3 | GTF3C4, POLR3K, POLR3H |
| 27 | 1.5 | 3 | 3 | ACER1, SGPP1, SMPD2 |
| 28 | 1.5 | 3 | 3 | DHFR, CBS, TYMS |
| 29 | 1.5 | 3 | 3 | ABCC2, SLC35A2, CYP2D6 |
| 30 | 1.5 | 3 | 3 | DPH3, KTI12, ELP2 |
| 31 | 1.5 | 3 | 3 | RBMS2, EPDR1, DIS3L2 |
| 32 | 1.5 | 3 | 3 | DTX3L, TRIM59, C3orf58 |
| 33 | 1.5 | 3 | 3 | PNPLA4, RDH10, ADH5 |
| 34 | 1.5 | 3 | 3 | EPPIN, CST11, SPINT4 |
| 35 | 1.5 | 3 | 3 | IFT74, IFT43, WDR19 |
| 36 | 1.5 | 3 | 3 | SH3BGRL3, COA3, RHBDF2 |
| 37 | 1.5 | 3 | 3 | MMP28, MMP17, MATN2 |
| 38 | 1.5 | 3 | 3 | FAM183B, CD163L1, FAM109B |
