# Supplementary material for: A multidimensional integration analysis reveals potential bridging targets in the process of colorectal cancer liver metastasis
Source: PLoS One. 2017 Jun 19;12(6):e0178760. doi: 10.1371/journal.pone.0178760 (PMC5476238; doi:10.1371/journal.pone.0178760)
Supplement: S5 Table — (DOCX) [file pone.0178760.s005.docx]

**Supplemental Table 5: Modules with significant overlap**

| RMCT module | LMCT module | Overlapping P value |
| --- | --- | --- |
| RMCT-2 | LMCT-2 | 0.01718014 |
| RMCT-6 | LMCT-12 | 0.01504767 |
| RMCT-6 | LMCT-6 | 0.01640131 |
