# Supplementary material for: A multidimensional integration analysis reveals potential bridging targets in the process of colorectal cancer liver metastasis
Source: PLoS One. 2017 Jun 19;12(6):e0178760. doi: 10.1371/journal.pone.0178760 (PMC5476238; doi:10.1371/journal.pone.0178760)
Supplement: S6 Table — (DOCX) [file pone.0178760.s006.docx]

**Supplemental Table 6: The function of modules with significant overlap**

| Module | GO Term | P Value |
| --- | --- | --- |
| PMCT-2 | GO:0007186~G-protein coupled receptor protein signaling pathway | 3.46E-15 |
|  | GO:0007166~cell surface receptor linked signal transduction | 9.57E-12 |
|  | GO:0019932~second-messenger-mediated signaling | 1.84E-08 |
|  | GO:0007610~behavior | 2.02E-06 |
|  | GO:0009611~response to wounding | 4.55E-06 |
|  | GO:0007204~elevation of cytosolic calcium ion concentration | 1.46E-05 |
|  | GO:0051480~cytosolic calcium ion homeostasis | 1.93E-05 |
|  | GO:0006954~inflammatory response | 6.84E-05 |
|  | GO:0006874~cellular calcium ion homeostasis | 1.07E-04 |
|  | GO:0008015~blood circulation | 1.14E-04 |
|  | GO:0003013~circulatory system process | 1.14E-04 |
|  | GO:0055074~calcium ion homeostasis | 1.19E-04 |
|  | GO:0006875~cellular metal ion homeostasis | 1.40E-04 |
|  | GO:0006952~defense response | 1.41E-04 |
|  | GO:0055065~metal ion homeostasis | 1.66E-04 |
|  | GO:0048015~phosphoinositide-mediated signaling | 2.39E-04 |
|  | GO:0030005~cellular di-, tri-valent inorganic cation homeostasis | 2.45E-04 |
|  | GO:0055066~di-, tri-valent inorganic cation homeostasis | 2.99E-04 |
|  | GO:0030003~cellular cation homeostasis | 3.77E-04 |
|  | GO:0055080~cation homeostasis | 5.90E-04 |
|  | GO:0050878~regulation of body fluid levels | 9.50E-04 |
|  | GO:0007242~intracellular signaling cascade | 0.001082 |
|  | GO:0006935~chemotaxis | 0.001369 |
|  | GO:0042330~taxis | 0.001369 |
|  | GO:0006873~cellular ion homeostasis | 0.001603 |
|  | GO:0055082~cellular chemical homeostasis | 0.001699 |
|  | GO:0050801~ion homeostasis | 0.002225 |
|  | GO:0007200~activation of phospholipase C activity by G-protein coupled receptor protein signaling pathway coupled to IP3 second messenger | 0.002377 |
|  | GO:0003018~vascular process in circulatory system | 0.002948 |
|  | GO:0019725~cellular homeostasis | 0.003569 |
|  | GO:0010863~positive regulation of phospholipase C activity | 0.003688 |
|  | GO:0007202~activation of phospholipase C activity | 0.003688 |
|  | GO:0010518~positive regulation of phospholipase activity | 0.004145 |
|  | GO:0010517~regulation of phospholipase activity | 0.004384 |
|  | GO:0060193~positive regulation of lipase activity | 0.004879 |
|  | GO:0048878~chemical homeostasis | 0.004996 |
|  | GO:0007626~locomotory behavior | 0.006263 |
|  | GO:0060191~regulation of lipase activity | 0.006512 |
|  | GO:0007596~blood coagulation | 0.008855 |
|  | GO:0050817~coagulation | 0.008855 |
|  | GO:0007599~hemostasis | 0.009883 |
| PMCT-6 | GO:0022904~respiratory electron transport chain | 5.00E-08 |
|  | GO:0045333~cellular respiration | 4.08E-07 |
|  | GO:0022900~electron transport chain | 9.14E-07 |
|  | GO:0042775~mitochondrial ATP synthesis coupled electron transport | 1.82E-06 |
|  | GO:0042773~ATP synthesis coupled electron transport | 1.82E-06 |
|  | GO:0015980~energy derivation by oxidation of organic compounds | 2.90E-06 |
|  | GO:0006119~oxidative phosphorylation | 1.71E-05 |
|  | GO:0006120~mitochondrial electron transport, NADH to ubiquinone | 4.11E-05 |
|  | GO:0006091~generation of precursor metabolites and energy | 1.22E-04 |
|  | GO:0006793~phosphorus metabolic process | 6.37E-04 |
|  | GO:0006796~phosphate metabolic process | 6.37E-04 |
|  | GO:0016310~phosphorylation | 0.001382 |
|  | GO:0007167~enzyme linked receptor protein signaling pathway | 0.002047 |
|  | GO:0055114~oxidation reduction | 0.003118 |
|  | GO:0007517~muscle organ development | 0.004629 |
|  | GO:0007507~heart development | 0.004878 |
|  | GO:0007010~cytoskeleton organization | 0.004908 |
|  | GO:0042325~regulation of phosphorylation | 0.006208 |
|  | GO:0019220~regulation of phosphate metabolic process | 0.007141 |
|  | GO:0051174~regulation of phosphorus metabolic process | 0.007141 |
|  | GO:0007017~microtubule-based process | 0.007653 |
|  | GO:0070271~protein complex biogenesis | 0.008219 |
|  | GO:0006461~protein complex assembly | 0.008219 |
| LMCT-2 | GO:0007186~G-protein coupled receptor protein signaling pathway | 3.64E-11 |
|  | GO:0007166~cell surface receptor linked signal transduction | 4.71E-10 |
|  | GO:0019932~second-messenger-mediated signaling | 1.13E-07 |
|  | GO:0035150~regulation of tube size | 2.78E-07 |
|  | GO:0050880~regulation of blood vessel size | 2.78E-07 |
|  | GO:0003018~vascular process in circulatory system | 4.01E-07 |
|  | GO:0042310~vasoconstriction | 7.43E-07 |
|  | GO:0048015~phosphoinositide-mediated signaling | 2.16E-06 |
|  | GO:0006939~smooth muscle contraction | 5.90E-06 |
|  | GO:0007200~activation of phospholipase C activity by G-protein coupled receptor protein signaling pathway coupled to IP3 second messenger | 2.36E-05 |
|  | GO:0007267~cell-cell signaling | 2.64E-05 |
|  | GO:0014829~vascular smooth muscle contraction | 4.11E-05 |
|  | GO:0003013~circulatory system process | 4.20E-05 |
|  | GO:0008015~blood circulation | 4.20E-05 |
|  | GO:0010863~positive regulation of phospholipase C activity | 4.62E-05 |
|  | GO:0007202~activation of phospholipase C activity | 4.62E-05 |
|  | GO:0010518~positive regulation of phospholipase activity | 5.53E-05 |
|  | GO:0010517~regulation of phospholipase activity | 6.02E-05 |
|  | GO:0060193~positive regulation of lipase activity | 7.10E-05 |
|  | GO:0007610~behavior | 1.10E-04 |
|  | GO:0060191~regulation of lipase activity | 1.11E-04 |
|  | GO:0043085~positive regulation of catalytic activity | 1.79E-04 |
|  | GO:0007242~intracellular signaling cascade | 1.93E-04 |
|  | GO:0044057~regulation of system process | 2.99E-04 |
|  | GO:0044093~positive regulation of molecular function | 3.13E-04 |
|  | GO:0006936~muscle contraction | 5.84E-04 |
|  | GO:0014032~neural crest cell development | 5.94E-04 |
|  | GO:0014033~neural crest cell differentiation | 5.94E-04 |
|  | GO:0032101~regulation of response to external stimulus | 6.53E-04 |
|  | GO:0003012~muscle system process | 7.67E-04 |
|  | GO:0051345~positive regulation of hydrolase activity | 9.22E-04 |
|  | GO:0042127~regulation of cell proliferation | 0.001211 |
|  | GO:0014031~mesenchymal cell development | 0.001418 |
|  | GO:0048762~mesenchymal cell differentiation | 0.001418 |
|  | GO:0060485~mesenchyme development | 0.001474 |
|  | GO:0032103~positive regulation of response to external stimulus | 0.002223 |
|  | GO:0046883~regulation of hormone secretion | 0.002363 |
|  | GO:0007631~feeding behavior | 0.002579 |
|  | GO:0046903~secretion | 0.004031 |
|  | GO:0014826~vein smooth muscle contraction | 0.004428 |
|  | GO:0008217~regulation of blood pressure | 0.005335 |
|  | GO:0051336~regulation of hydrolase activity | 0.00558 |
|  | GO:0007204~elevation of cytosolic calcium ion concentration | 0.00642 |
|  | GO:0051480~cytosolic calcium ion homeostasis | 0.007355 |
|  | GO:0006873~cellular ion homeostasis | 0.007448 |
|  | GO:0055082~cellular chemical homeostasis | 0.007782 |
|  | GO:0050801~ion homeostasis | 0.009521 |
| LMCT-6 | GO:0022900~electron transport chain | 2.34E-12 |
|  | GO:0006091~generation of precursor metabolites and energy | 1.27E-09 |
|  | GO:0055114~oxidation reduction | 5.98E-07 |
|  | GO:0042775~mitochondrial ATP synthesis coupled electron transport | 1.29E-04 |
|  | GO:0042773~ATP synthesis coupled electron transport | 1.92E-04 |
|  | GO:0022904~respiratory electron transport chain | 2.95E-04 |
|  | GO:0045333~cellular respiration | 0.001558 |
|  | GO:0006119~oxidative phosphorylation | 0.002425 |
|  | GO:0015980~energy derivation by oxidation of organic compounds | 0.003194 |
|  | GO:0006412~translation | 0.004503 |
|  | GO:0006122~mitochondrial electron transport, ubiquinol to cytochrome c | 0.006246 |
| LMCT-12 | GO:0007018~microtubule-based movement | 0.001209 |
|  | GO:0007017~microtubule-based process | 0.004545 |
