# Supplementary material for: A multidimensional integration analysis reveals potential bridging targets in the process of colorectal cancer liver metastasis
Source: PLoS One. 2017 Jun 19;12(6):e0178760. doi: 10.1371/journal.pone.0178760 (PMC5476238; doi:10.1371/journal.pone.0178760)
Supplement: S7 Table — (DOCX) [file pone.0178760.s007.docx]

**Supplemental Table 7: Modules with significant crosstalk**

| LMCT module | Crosstalk edge | PMCT module | P value |
| --- | --- | --- | --- |
| LMCT-10 | 13 | PMCT-9 | 0.02 |
| LMCT-7 | 36 | PMCT-5 | 0.006 |
| LMCT-24 | 7 | PMCT-6 | 0.033 |
