# Supplementary material for: A multidimensional integration analysis reveals potential bridging targets in the process of colorectal cancer liver metastasis
Source: PLoS One. 2017 Jun 19;12(6):e0178760. doi: 10.1371/journal.pone.0178760 (PMC5476238; doi:10.1371/journal.pone.0178760)
Supplement: S8 Table — (DOCX) [file pone.0178760.s008.docx]

**Supplemental Table 8: The function of modules with significant crosstalk**

| Module | GO Term | P Value |
| --- | --- | --- |
| PMCT-5 | GO:0006334~nucleosome assembly | 8.18E-12 |
|  | GO:0031497~chromatin assembly | 9.79E-12 |
|  | GO:0065004~protein-DNA complex assembly | 1.23E-11 |
|  | GO:0034728~nucleosome organization | 1.38E-11 |
|  | GO:0006323~DNA packaging | 4.44E-11 |
|  | GO:0006333~chromatin assembly or disassembly | 6.74E-11 |
|  | GO:0034622~cellular macromolecular complex assembly | 6.96E-09 |
|  | GO:0034621~cellular macromolecular complex subunit organization | 1.25E-08 |
|  | GO:0006325~chromatin organization | 1.66E-08 |
|  | GO:0051276~chromosome organization | 5.81E-08 |
|  | GO:0065003~macromolecular complex assembly | 2.83E-07 |
|  | GO:0043933~macromolecular complex subunit organization | 3.93E-07 |
| PMCT-6 | GO:0022904~respiratory electron transport chain | 5.00E-08 |
|  | GO:0045333~cellular respiration | 4.08E-07 |
|  | GO:0022900~electron transport chain | 9.14E-07 |
|  | GO:0042775~mitochondrial ATP synthesis coupled electron transport | 1.82E-06 |
|  | GO:0042773~ATP synthesis coupled electron transport | 1.82E-06 |
|  | GO:0015980~energy derivation by oxidation of organic compounds | 2.90E-06 |
|  | GO:0006119~oxidative phosphorylation | 1.71E-05 |
|  | GO:0006120~mitochondrial electron transport, NADH to ubiquinone | 4.11E-05 |
|  | GO:0006091~generation of precursor metabolites and energy | 1.22E-04 |
|  | GO:0006793~phosphorus metabolic process | 6.37E-04 |
|  | GO:0006796~phosphate metabolic process | 6.37E-04 |
|  | GO:0016310~phosphorylation | 0.001382 |
|  | GO:0007167~enzyme linked receptor protein signaling pathway | 0.002047 |
|  | GO:0055114~oxidation reduction | 0.003118 |
|  | GO:0007517~muscle organ development | 0.004629 |
|  | GO:0007507~heart development | 0.004878 |
|  | GO:0007010~cytoskeleton organization | 0.004908 |
|  | GO:0042325~regulation of phosphorylation | 0.006208 |
|  | GO:0019220~regulation of phosphate metabolic process | 0.007141 |
|  | GO:0051174~regulation of phosphorus metabolic process | 0.007141 |
|  | GO:0007017~microtubule-based process | 0.007653 |
|  | GO:0006461~protein complex assembly | 0.008219 |
|  | GO:0070271~protein complex biogenesis | 0.008219 |
| PMCT-9 | GO:0008284~positive regulation of cell proliferation | 0.005381 |
| LMCT-7 | GO:0006334~nucleosome assembly | 5.54E-14 |
|  | GO:0031497~chromatin assembly | 7.70E-14 |
|  | GO:0065004~protein-DNA complex assembly | 1.17E-13 |
|  | GO:0034728~nucleosome organization | 1.43E-13 |
|  | GO:0006323~DNA packaging | 1.19E-12 |
|  | GO:0006333~chromatin assembly or disassembly | 2.53E-12 |
|  | GO:0034622~cellular macromolecular complex assembly | 4.04E-10 |
|  | GO:0034621~cellular macromolecular complex subunit organization | 1.24E-09 |
|  | GO:0051276~chromosome organization | 2.35E-08 |
|  | GO:0065003~macromolecular complex assembly | 3.72E-08 |
|  | GO:0006325~chromatin organization | 4.08E-08 |
|  | GO:0043933~macromolecular complex subunit organization | 7.29E-08 |
|  | GO:0031399~regulation of protein modification process | 2.57E-05 |
|  | GO:0032268~regulation of cellular protein metabolic process | 3.50E-04 |
|  | GO:0051439~regulation of ubiquitin-protein ligase activity during mitotic cell cycle | 4.13E-04 |
|  | GO:0051438~regulation of ubiquitin-protein ligase activity | 5.45E-04 |
|  | GO:0031401~positive regulation of protein modification process | 5.58E-04 |
|  | GO:0051340~regulation of ligase activity | 6.08E-04 |
|  | GO:0009968~negative regulation of signal transduction | 0.001043 |
|  | GO:0031396~regulation of protein ubiquitination | 0.001123 |
|  | GO:0032270~positive regulation of cellular protein metabolic process | 0.001269 |
|  | GO:0010604~positive regulation of macromolecule metabolic process | 0.00146 |
|  | GO:0051247~positive regulation of protein metabolic process | 0.001481 |
|  | GO:0010648~negative regulation of cell communication | 0.001597 |
|  | GO:0031400~negative regulation of protein modification process | 0.001852 |
|  | GO:0022604~regulation of cell morphogenesis | 0.002436 |
|  | GO:0043085~positive regulation of catalytic activity | 0.003885 |
|  | GO:0022402~cell cycle process | 0.005525 |
|  | GO:0048729~tissue morphogenesis | 0.005943 |
|  | GO:0032269~negative regulation of cellular protein metabolic process | 0.005943 |
|  | GO:0044093~positive regulation of molecular function | 0.006439 |
|  | GO:0051248~negative regulation of protein metabolic process | 0.006604 |
|  | GO:0000278~mitotic cell cycle | 0.006706 |
|  | GO:0051603~proteolysis involved in cellular protein catabolic process | 0.007104 |
|  | GO:0044257~cellular protein catabolic process | 0.007253 |
|  | GO:0030879~mammary gland development | 0.007463 |
|  | GO:0051436~negative regulation of ubiquitin-protein ligase activity during mitotic cell cycle | 0.007928 |
|  | GO:0031145~anaphase-promoting complex-dependent proteasomal ubiquitin-dependent protein catabolic process | 0.007928 |
|  | GO:0030163~protein catabolic process | 0.008247 |
|  | GO:0051444~negative regulation of ubiquitin-protein ligase activity | 0.008406 |
|  | GO:0051352~negative regulation of ligase activity | 0.008406 |
|  | GO:0051437~positive regulation of ubiquitin-protein ligase activity during mitotic cell cycle | 0.00865 |
|  | GO:0010720~positive regulation of cell development | 0.008897 |
|  | GO:0051443~positive regulation of ubiquitin-protein ligase activity | 0.009147 |
|  | GO:0051351~positive regulation of ligase activity | 0.009916 |
| LMCT-10 | GO:0002697~regulation of immune effector process | 7.97E-07 |
|  | GO:0010557~positive regulation of macromolecule biosynthetic process | 1.01E-04 |
|  | GO:0031328~positive regulation of cellular biosynthetic process | 1.34E-04 |
|  | GO:0009891~positive regulation of biosynthetic process | 1.47E-04 |
|  | GO:0032653~regulation of interleukin-10 production | 1.64E-04 |
|  | GO:0002694~regulation of leukocyte activation | 1.92E-04 |
|  | GO:0050865~regulation of cell activation | 2.35E-04 |
|  | GO:0015031~protein transport | 2.60E-04 |
|  | GO:0045184~establishment of protein localization | 2.75E-04 |
|  | GO:0010628~positive regulation of gene expression | 4.24E-04 |
|  | GO:0010604~positive regulation of macromolecule metabolic process | 5.33E-04 |
|  | GO:0045935~positive regulation of nucleobase, nucleoside, nucleotide and nucleic acid metabolic process | 6.20E-04 |
|  | GO:0008104~protein localization | 6.34E-04 |
|  | GO:0051173~positive regulation of nitrogen compound metabolic process | 7.33E-04 |
|  | GO:0045321~leukocyte activation | 8.01E-04 |
|  | GO:0050863~regulation of T cell activation | 0.001118 |
|  | GO:0001775~cell activation | 0.001508 |
|  | GO:0051249~regulation of lymphocyte activation | 0.002194 |
|  | GO:0002700~regulation of production of molecular mediator of immune response | 0.002371 |
|  | GO:0048663~neuron fate commitment | 0.002487 |
|  | GO:0045941~positive regulation of transcription | 0.002721 |
|  | GO:0030111~regulation of Wnt receptor signaling pathway | 0.002977 |
|  | GO:0045944~positive regulation of transcription from RNA polymerase II promoter | 0.003829 |
|  | GO:0001817~regulation of cytokine production | 0.003877 |
|  | GO:0046649~lymphocyte activation | 0.005054 |
|  | GO:0002703~regulation of leukocyte mediated immunity | 0.005179 |
|  | GO:0042129~regulation of T cell proliferation | 0.005346 |
|  | GO:0045619~regulation of lymphocyte differentiation | 0.005515 |
|  | GO:0008284~positive regulation of cell proliferation | 0.005651 |
|  | GO:0042035~regulation of cytokine biosynthetic process | 0.007537 |
|  | GO:0006357~regulation of transcription from RNA polymerase II promoter | 0.008005 |
|  | GO:0045893~positive regulation of transcription, DNA-dependent | 0.009259 |
|  | GO:0050670~regulation of lymphocyte proliferation | 0.009404 |
|  | GO:0051254~positive regulation of RNA metabolic process | 0.009529 |
|  | GO:0070663~regulation of leukocyte proliferation | 0.009623 |
|  | GO:0032944~regulation of mononuclear cell proliferation | 0.009623 |
|  | GO:0051276~chromosome organization | 0.009804 |
